# Development of a DNA microarray assay for rapid detection of fifteen bacterial pathogens in pneumonia

**DOI:** 10.1186/s12866-020-01842-3

**Published:** 2020-06-23

**Authors:** Xiuqing Ma, Yanqin Li, Yuan Liang, Yang Liu, Ling Yu, Chunsun Li, Qiqi Liu, Liangan Chen

**Affiliations:** 1 Department of Pulmonary & Critical Care Medicine, Chinese PLA General Hospital, Beijing, China; 2 Department of Biotechnology, Beijing Institute of Radiation Medicine, Beijing, China

**Keywords:** DNA microarray, Bacteria, Pneumonia

## Abstract

**Background:**

The rapid identification of pathogenic bacteria is important for determining an appropriate antimicrobial therapy for pneumonia, but traditional bacterial culture is time-consuming and labourious. The aim of this study was to develop and evaluate a DNA microarray assay for the simultaneous detection of fifteen bacterial species directly from respiratory tract specimens in patients with pneumonia. These species included *Streptococcus pneumoniae, Staphylococcus aureus, Haemophilus influenzae, Escherichia coli, Klebsiella pneumoniae, Pseudomonas aeruginosa, Acinetobacter baumannii, Mycoplasma pneumoniae, Enterococcus faecalis, Enterococcus faecium, Enterobacter cloacae, Stenotrophomonas maltophilia, Burkholderia cepacia, Legionella pneumophila* and *Chlamydia pneumoniae.* The 16S rDNA genes and other specific genes of each pathogen were chosen as the amplification targets, amplified via multiplex polymerase chain reaction (PCR), and hybridized to oligonucleotide probes in a microarray.

**Results:**

The DNA microarray detection limit was 10^3^ copies/μL. Nineteen standard strains and 119 clinical isolates were correctly detected with our microarray, and 3 nontarget species from 4 clinical isolates were not detected. Additionally, bacterial pathogens were accurately identified when two or three bacterial targets were mixed together. Furthermore, the results for 99.4% (156/157) of clinical specimens were the same as those from a conventional assay.

**Conclusions:**

We developed a DNA microarray that could simultaneously detect various bacterial pathogens in pneumonia. The method described here has the potential to provide considerable labour and time savings due to its ability to screen for 15 bacterial pathogens simultaneously.

## Background

The rapid identification of pathogenic bacteria is important for selecting an appropriate antimicrobial therapy for pneumonia [1]. However, current standard microbiological culture-based tests are labourious and time consuming [2]. Patients often receive empirical broad-spectrum antimicrobial treatment while waiting for microbiology results. Hence, novel diagnostic approaches are urgently needed to improve early antimicrobial therapy for pneumonia.

Standard European guidelines for the diagnosis and management of pneumonia note that molecular diagnosis is a promising method for rapidly detecting pathogens [3]. Several molecular methods based on polymerase chain reaction (PCR) have been developed to detect species-specific genes. Such methods have been developed for the identification of *Pseudomonas aeruginosa* by the amplification of the specific exotoxin A gene [4], the identification of *Mycoplasma pneumoniae* using a fragment of the gene encoding P1 cytadhesin protein [5], the identification of *Haemophilus influenzae* by amplifying a fragment of the gene encoding the P6 outer membrane protein [6], and many others [7]. However, these methods have a narrow diagnostic spectrum.

To address this problem, multiplex PCR or ribosomal DNA (rDNA) has been used [8–10]. Although multiplex PCR can simultaneously detect several different bacteria, the number of bacteria is still limited within a single test. 16S rDNA sequences exist universally within bacteria and include both conserved regions and species-specific regions [11]. The most common method is to use a universal primer pair to amplify species-specific fragments of 16S rDNA. However, it is not possible to achieve complete discrimination among some genera, such as *Enterobacteriaceae*, in which the 16S rDNA sequences of *Klebsiella pneumoniae, Enterobacter cloacae* and *Escherichia coli* are very similar [12].

To extend the detection spectrum and shorten the detection time, we developed a DNA microarray assay that can detect 15 bacterial respiratory pathogens associated with pneumonia, including *Streptococcus pneumoniae, Staphylococcus aureus, Haemophilus influenzae, Escherichia coli, Klebsiella pneumoniae, Pseudomonas aeruginosa, Acinetobacter baumannii, Mycoplasma pneumoniae, Enterococcus faecalis, Enterococcus faecium, Enterobacter cloacae, Stenotrophomonas maltophilia, Burkholderia cepacia, Legionella pneumophila* and *Chlamydia pneumoniae*. To identify bacteria at the species level, we chose to use a 16S rDNA probe combined with a species–specific probe to detect each bacterium. The sequences of the species–specific probes corresponded to 15 species–specific genes.

## Results

### Primer design and evaluation

Specific genes for the targeting of the 15 different bacterial species were selected based on a thorough literature search for particular bacterial housekeeping genes. The 15 bacterial-specific genes were *lytA* of *Streptococcus pneumoniae* [8]*, nuc* of *Staphylococcus aureus* [8]*, P6* of *Haemophilus influenzae* [13]*, phoA* of *Escherichia coli* [14]*, mdh* of *Klebsiella pneumoniae* [14]*, toxA* of *Pseudomonas aeruginosa* [4]*, gltA* of *Acinetobacter baumannii* [14]*, P1* of *Mycoplasma pneumoniae* [5]*, ddl* of *Enterococcus faecalis* and *Enterococcus faecium* [15]*, dnaJ* of *Enterobacter cloacae* [16]*, chitA* of *Stenotrophomonas maltophilia* [17]*, recA* of *Burkholderia cepacia* [18]*, mip* of *Legionella pneumophila* and *ompA* of *Chlamydia pneumoniae* [5, 19]*.* We designed all primers in house. Three pairs of primers were initially designed for each specific gene, and the primer pairs were checked by BLAST searches (http://www.ncbi.nih.gov). If all 3 pairs of primers failed to be successfully amplified, we designed 3 alternative pairs of primers. After repeated screening, 16 pairs of primers, including one pair of universal 16S rDNA primers and 15 pairs of bacterial-specific gene primers, were selected and successfully amplified (Table 1). All primers included in an individual group for multiplex asymmetric PCR presented a similar melting temperature. The specificity of the 16 paired primers was preliminarily tested by PCR, and the PCR products were examined by 2% agarose gel electrophoresis (Fig. S1). All primers and probes were finally confirmed by sequence analysis of the PCR products from the reference plasmids.

**Table 1:** Oligonucleotide sequences

Assay	Organism	Gene target	Primer (5’-3’)	Probe (5’-3’)	Product (bp)
Multiplex asymmetric PCR 1	*Acinetobacter baumannii*	Citrate synthase(*gltA*)	F:CTCTGCTGGTATCTCTGCTCT	TGTTGCTGAGTTCATGGAAAAAGTTAAACG-TTTTTTTTTTTT-amino	222
			R:Biotin-TGCTTCAAGAACTTCGTCAC		
	*Staphylococcus aureus*	Thermostable nuclease (*nuc*)	F:AGCGATTGATGGTGATAC	CAAAGAACTGATAAATATGGACGTGGC-TTTTTTTTTTTT-amino	276
			R: Biotin-AAGCCTTGACGAACTAAAG		
	*Enterococcus faecalis*	D-alanine--D-alanine ligase (*ddl*)	F:GAACGACCACAAAATAAAG	TTACATGGGCCAAATGGTGAAGATGGAACA-TTTTTTTTTTTT-amino	275
			R: Biotin-GCCAACAGTTTGTAAAAGAT		
	*Mycoplasma pneumoniae*	Adhesin protein (*P1*)	F:TGGTCCTACACCGACTTACA	TGAGGTGAATGGGTTGTTGAATCCG-TTTTTTTTTTTT-amino	107
			R: Biotin-TTCCCAAAATAGGTTTCCAC		
Multiplex asymmetric PCR 2	*Enterobacter cloacae*	Molecular chaperone (*dnaJ*)	F:GTCACCAAAGAGATCCGTA	GCAGGCGATCTGTACGTTCAGG-TTTTTTTTTTTT-amino	524
			R: Biotin-CGCATGCGGAACAGCTT		
	*Burkholderia cepacia*	Recombinase A-like (*recA*)	F:ATATCCAGGTCGTCTCCA	TGGTGCGCTCGGGCTCGATCGACA-TTTTTTTTTTTT-amino	453
			R: Biotin-AGTTCGTGCGCTTGATCGT		
	*Klebsiella pneumoniae*	Malate dehydrogenase (*mdh*)	F:GCGTGGCGGTAGATCTAAGTCATA	AAAGCCGGCGTGTACGATAA-TTTTTTTTTTTT-amino	364
			R: Biotin-TTCAGCTCCGCCACAAAGGTA		
	*Escherichia coli*	Bacterial alkaline phosphatase (*phoA*)	F:CCAACGATTCTGGAAATGG	CGCCAAATCCGCAACGTAATGACAGTGTACCAACCC-TTTTTTTTTTTT-amino	527
			R: Biotin-CAATGGCTTTGTCGGTCAT		
	*Pseudomonas aeruginosa*	Exotoxin A (*toxA*)	F:TCATCCACGAACTGAACG	TTGTGCCTGCTCGACCCGCTGGACGGGGTCTACAACTACCTCGCCCAG-TTTTTTTTTTTT-amino	325
			R: Biotin-ATCTTGCCTTCCCAGGTAT		
	*Stenotrophomonas maltophilia*	Chitinase A (*chitA*)	F:TCAAGCAGCTCAAGGCCAA	TACCACCCGTACCTGGAC-TTTTTTTTTTTT-amino	435
			R: Biotin-TGGAAGTCGTAGGTCATC		
Multiplex asymmetric PCR 3	*Haemophilus infuenzae*	Adhesin protein (*P6*)	F:TCTAACAACGATGCTGCAGG	GAACGTGGTACACCAGAATACAACATCGC-TTTTTTTTTTTT-amino	296
			R: Biotin-CCAGCATCAACACCTTTACC		
	*Legionella pneumophila*	Macrophage infectivity potentiator (*mip*)	F: CTACAGACAAGGATAAGT	ATAGCATTGGTGCCGATTTGGGGAAGAA-TTTTTTTTTTTT-amino	108
			R: Biotin-CTTGCATGCCTTTAGCCA		
	*Enterococcus faecium*	D-alanine--D-alanine ligase (*ddl*)	F:GCTAAAGCCACGCCTTCTA	TCCTTTTTCCGTCATCAGTATAAAGTATAG-TTTTTTTTTTTT-amino	264
			R: Biotin-GGTGACGGATGGAAATGTT		
	*Chlamydia pneumoniae*	Major outer membrane protein (*ompA*)	F:GATCCGCTGCTGCAAACTATACT	ACTGCCGTAGATAGACCTAACCCGGCCTA-TTTTTTTTTTTT-amino	85
			R:Biotin-GTGAACCACTCTGCATCGTGTAA		
	*Streptococcus pneumoniae*	N-acetylmuramoyl-L-alanine amidase (*lytA*)	F: CCATCTGGCTCTACTGTGAA	CAAAGTAGTACCAAGTGCCATTGATTTTC-TTTTTTTTTTTT-amino	433
			R: Biotin-GAGAACGGCTTGACGATT		
	Bacteria (universal primers)	16S rDNA	F:AGAGTTTGATCMTGGCTCAG(M=A/C)		575
			R: Biotin-CGTATTACCGCGGCTGCTG		
	*Acinetobacter* spp.	16S rDNA		CCTAGAGATAGTGGACGTTAC-TTTTTTTTTTTT-amino	
	*Staphylococcus* spp.	16S rDNA		ACATATGTGTAAGTAACTGTGCACATCTTGACGGTA-TTTTTTTTTTTT-amino	
	*Enterococcus faecalis*	16S rDNA		AGTGCTTGCACTCAATTGGAAAGAGGAGTGG-TTTTTTTTTTTT-amino	
	*Mycoplasma* spp.	16S rDNA		GACCTGCAAGGGTTCGT-TTTTTTTTTTTT-amino	
	*Burkholderia* spp.	16S rDNA		TTGGCTCTAATACAGTCGG-TTTTTTTTTTTT-amino	
	*Klebsiella spp./Escherichia spp./Enterobacter* spp*.*	16S rDNA		GGTTAATAACCTCATCGATTGACGTTACCCTGC-TTTTTTTTTTTT-amino	
	*Pseudomonas* spp*.*	16S rDNA		TTGCTGTTTTGACGTTAC-TTTTTTTTTTTT-amino	
	*Stenotrophomonas* spp*.*	16S rDNA		CCAGCTGGTTAATACCCGGTTGGGA-TTTTTTTTTTTT-amino	
	*Haemophilus* spp.	16S rDNA		GAGGAAGGTTGATGTGTTA-TTTTTTTTTTTT-amino	
	*Legionella* spp.	16S rDNA		AGGGTTGATAGGTTAAGAGCTGATTAA-TTTTTTTTTTTT-amino	
	*Enterococcus faecium*	16S rDNA		CAAGGATGAGAGTAACTGTTCATCCC-TTTTTTTTTTTT-amino	
	*Chlamydia* spp.	16S rDNA		CCGAATGTAGTGTAATTAGGC-TTTTTTTTTTTT-amino	
	*Streptococcus* spp*.*	16S rDNA		TGTGAGAGTGGAAAGTTCACACTG-TTTTTTTTTTTT-amino	
Positive control	Bacteria	16S rDNA		ACTCCTACGGGAGGCAGCAG-TTTTTTTTTTTT-amino	
Negetive control 1	Human	Epidermal growth factor receptor (EGFR)		TCAGAGCCTGTGTTTCTACCAA-TTTTTTTTTTTT-amino	
Negetive control 2	Virus	Neuraminidase (NA)		CATCAATAGGGTCCGATA-TTTTTTTTTTTT-amino	
Negetive control 3	Fungus	Internal transcribed spacer2 (ITS2)		CGAACGCAAATCAATCTTTTTCCAGGT-TTTTTTTTTTTT-amino	
Quality control^a^		20T		Biotin-TTTTTTTTTTTTTTTTTTTT-amino	

*F* Forward, *R* Reverse

^a^Repeat sequence of 20T with an amino-labeled 3’-end, Biotin-labeled 5’-end was used as microarray quality control

### The limit of detection and accuracy of the microarray

**Fig. 1:**
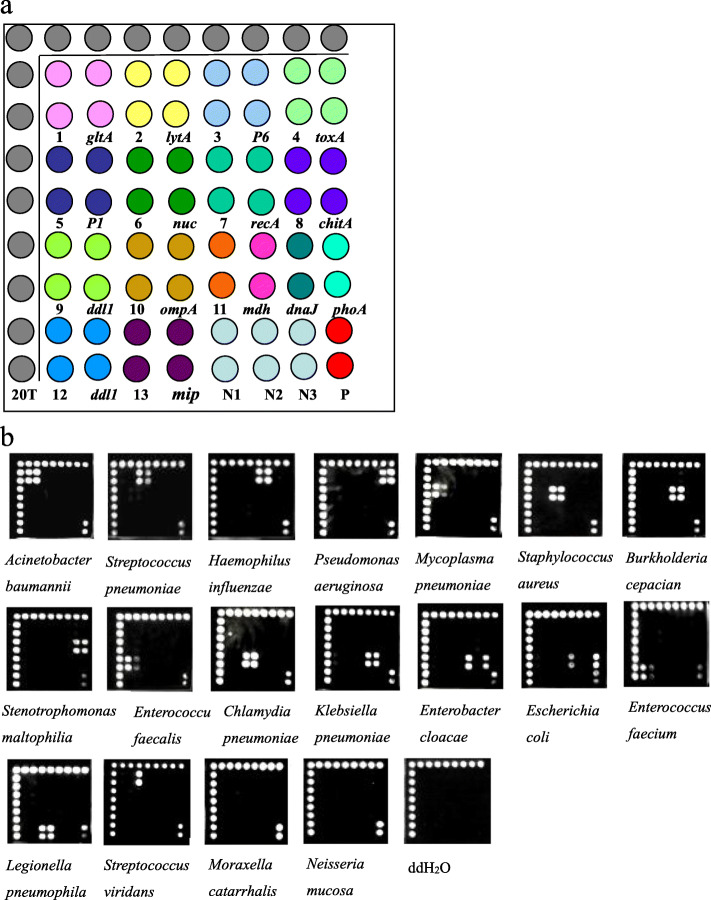
**a.** The layout of the hybridization capture-chip. The probe 20 T is the QC probe. The probe N1, N2, N3 are the negative control probes. The probe P is the universal 16S rDNA probe. Each probe was spotted as two. The sequences of probe 1–13 all come from 16S rDNA and their corresponding target pathogen were: 1 *Acinetobacter baumannii*; 2 *Streptococcus pneumoniae*; 3 *Haemophilus influenzae*; 4 *Pseudomonas aeruginosa*; 5 *Mycoplasma pneumoniae*; 6 *Staphylococcus aureus*; 7 *Burkholderia cepacia*; 8 *Stenotrophomonas maltophilia*; 9 *Enterococcus faecalis*; 10 *Chlamydia pneumoniae*; 11 *Klebsiella pneumoniae* or *Enterobacter cloacae* or *Escherichia coli*; 12 *Enterococcus faecium*; 13 *Legionella pneumophila*, respectively. **b.** The typical hybridization results of fifteen species of bacterial pathogens in pneumonia, non-target bacteria from pure bacterial cultures and ddH2O

**Fig. 2:**
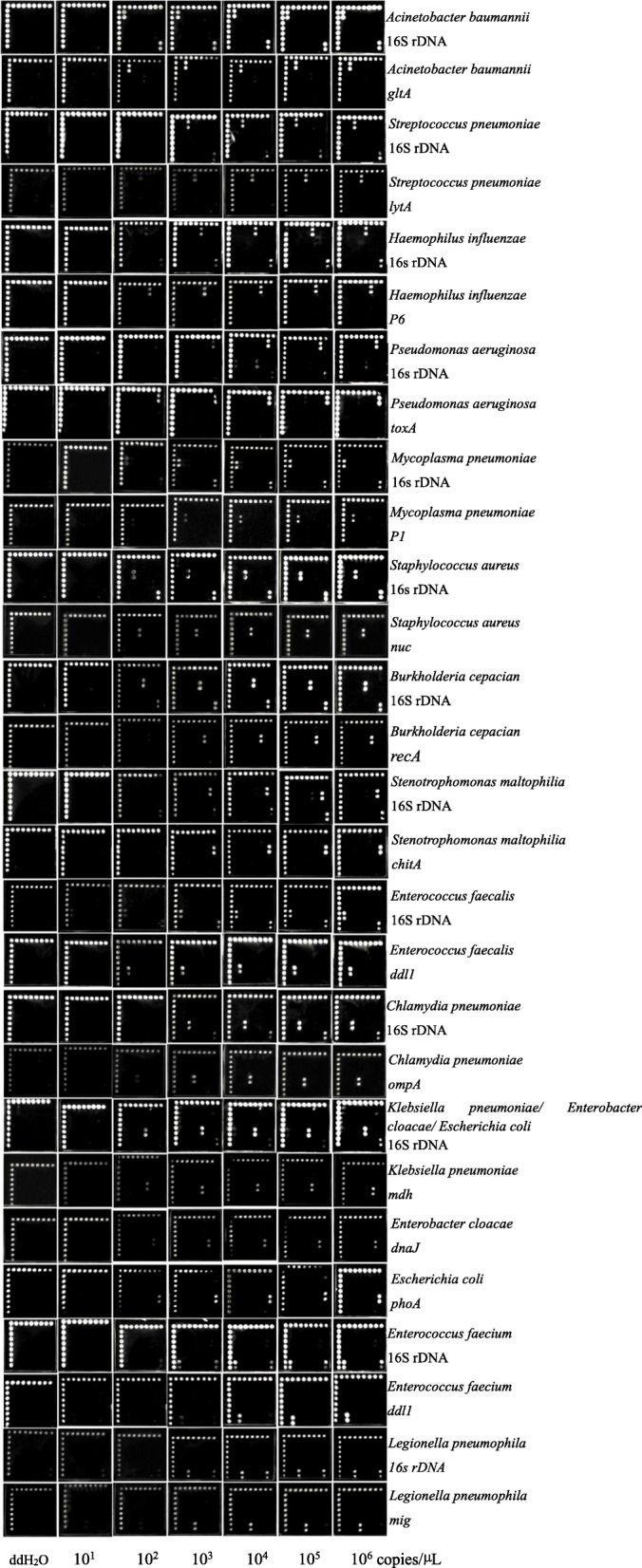
The sensitivity of the pathogen probes. Microarray hybridized with PCR products which diluted for concentration gradient. 10 μL dilution used in each well, and the concentration of probes were 50 μM

**Fig. 3:**
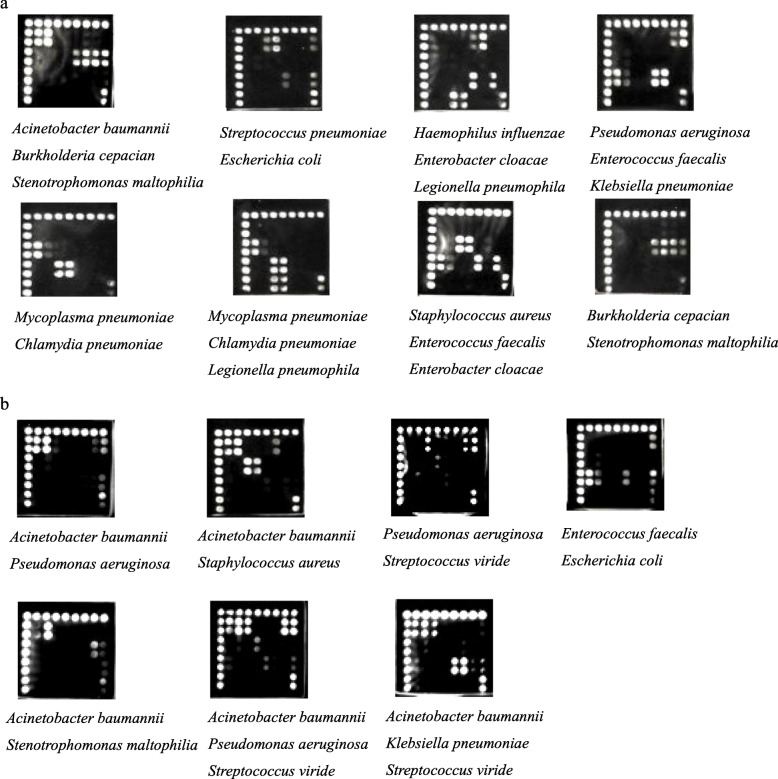
The specificity of the pathogen probes. a Microarray hybridized with PCR products amplified from mixed plasmid DNAs. b The hybridization results of clinical samples which contains two or more target pathogens

The microarray layout is shown in Fig. 1a. The detection limit of each probe reached 10^3^ copies/μL (Fig. 2). Positive diagnostic hybridization was confirmed only when three probes produced signals simultaneously. These three probes were the positive control probe from the conserved 16S rDNA sequence, the specific probe for the 16S rDNA sequence each target bacterium and the specific probe for the specific gene of each target bacterium. A total of 138 strains, including 19 standard strains and 119 clinical isolates (Table 2), were correctly detected with our microarray (Fig. 1b). Three nontarget bacterial species from 4 isolates in the collection were not detected (Fig. 1b). The hybridization signals emerged in order at the position corresponding to each target genus or species from the bacterial cultures, and none of the probes showed cross-hybridization between the target pathogens. For the 2 *Streptococcus viridans* isolates, we observed that only the specific 16S rDNA probe of *Streptococcus spp.* and the universal 16S rDNA probe produced signals. For one *Moraxella catarrhalis* isolate and one *Neisseria mucosa* isolate, a hybridization reaction only appeared at the position of the universal 16S rDNA probe. Furthermore, water was processed in parallel with the clinical samples as a negative PCR control, and the hybridization results showed no signal (Fig. 1b). In addition, all components within a mock specimen, which consisted of two or three target bacteria, could be accurately identified despite the presence of other components (Fig. 3a).

**Table 2:** Reference strains used in this study

Organism	No.	Source/strain
Target species		
*Acinetobacter baumannii*	25	ATCC19606; clinical isolates (24)
*Pseudomonas aeruginosa*	22	ATCC27853; clinical isolates (21)
*Klebsiella pneumoniae*	22	ATCC700603; clinical isolates (21)
*Enterococcus faecium*	17	ATCC35667; clinical isolates (16)
*Escherichia coli*	13	ATCC25922; clinical isolates (12)
*Enterococcus faecalis*	12	ATCC29212; clinical isolates (11)
*Mycoplasma pneumoniae*	5	ATCC29342; clinical isolates (4)
*Streptococcus pneumoniae*	4	ATCC49619; CMCC31001; clinical isolates (2)
*Staphylococcus aureus*	4	ATCC25923; N315; clinical isolates (2)
*Stenotrophomonas maltophilia*	4	CGMCC1.1788; clinical isolates (3)
*Burkholderia cepacia*	3	CGMCC1.1813; clinical isolates (2)
*Haemophilus infuenzae*	3	ATCC9006(serotypeA); ATCC33533(serotypeB); ATCC9007(serotypeC)
*Enterobacter cloacae*	2	ATCC13047; clinical isolate
*Legionella pneumophila*	1	ATCC33152
*Chlamydia pneumoniae*	1	ATCC VR1310
Non-target species		
*Streptococcus viridans*	2	Clinical isolates
*Moraxella catarrhalis*	1	Clinical isolate
*Neisseria mucosa*	1	Clinical isolate

*ATCC* American Type Culture Collection, *CMCC* National Center for Medical Culture Collections, *CGMCC* China General Microbiological Culture Collection Center

### Detection of clinical specimens

Among the 157 clinical specimens, 105 specimens exhibited only one pathogen, 36 specimens exhibited two pathogens, 5 specimens exhibited three pathogens, and 11 specimens exhibited no pathogens (Table 3). First, 151 bacterial pathogens belonging to 10 target species in clinical samples were correctly identified by the microarrays according to the results of bacterial culture. Second, the identification of one specimen with the microarray differed from the bacterial culture results. In scanning images of this specimen from two assays, only probes for *Acinetobacter baumannii* and the universal 16S rDNA probe presented a signal; therefore, we deduced that the specimen contained *Acinetobacter baumannii*. Additionally, the results for three replicates in the PCR analysis of the specimen based on the specific *nuc* gene of *Staphylococcus aureus* were negative. Finally, the microarray results for 40 bacterial pathogens belonging to 8 nontarget species in clinical samples were negative (Table 3). However, for *Streptococcus viridans, Staphylococcus hominis, Staphylococcus epidermidis* and *Staphylococcus haemolyticus*, specific 16S rDNA probes for these bacteria and the universal 16S rDNA probe exhibited signals, which indicated that this microarray could identify some nontarget bacteria at the genus level. For *Neisseria mucosa, Chryseobacterium indologenes, Ralstonia mannitolilytica,* and *Citrobacter freundii*, only the universal 16S rDNA probe presented a signal, which demonstrated that this microarray could determine whether the specimens contained bacteria. The hybridization results for clinical samples containing two or more target pathogens are shown in Fig. 3b.

**Table 3:** Identification results by microarray and bacterial culture of 157 clinical specimens

Hybridzation report	Number (%)	Microarray result	Standard culture
Correct identification	81 (39.9)	*Acinetobacter baumannii*	*Acinetobacter baumannii*
	29 (14.3)	*Pseudomonas aeruginosa*	*Pseudomonas aeruginosa*
	11 (5.4)	*Staphylococcus aureus*	*Staphylococcus aureus*
	11 (5.4)	*Klebsiella pneumoniae*	*Klebsiella pneumoniae*
	6 (3.0)	*Enterococcus faecium*	*Enterococcus faecium*
	5 (2.5)	*Stenotrophomonas maltophilia*	*Stenotrophomonas maltophilia*
	4 (2.0)	*Escherichia coli*	*Escherichia coli*
	2 (1.0)	*Burkholderia cepacia*	*Burkholderia cepacia*
	1 (0.5)	*Enterococcus faecalis*	*Enterococcus faecalis*
	1 (0.5)	*Streptococcus pneumoniae*	*Streptococcus pneumoniae*
	11 (5.4)	Negative	Negative
Incorrect identification			
Pathogens belong to our target	1 (0.5)	Negative	*Staphylococcus aureus*
Pathogens not belong to our target	29 (14.3)	Negative	*Streptococcus viridans*
	3 (1.5)	Negative	*Neisseria mucosa*
	2 (1.0)	Negative	*Staphylococcus hominis*
	2 (1.0)	Negative	*Staphylococcus epidermidis*
	1 (0.5)	Negative	*Staphylococcus haemolyticus*
	1 (0.5)	Negative	*Chryseobacterium indologenes*
	1 (0.5)	Negative	*Ralstonia mannitolilytica*
	1 (0.5)	Negative	*Citrobacter freundii*
	Total: 203		

## Discussion

We report the development of a novel DNA microarray for 15 important respiratory bacterial pathogens and the evaluation of its potential as a promising diagnostic tool for pneumonia. We employed two probes, one for a specific 16S rDNA sequence and the other for a specific gene sequence, to identify each target bacterium. The detection limit of each probe reached 10^3^ copies/μL. The detection accuracy of the microarray for the clinical isolates and specimens reached 100 and 99.4%, respectively.

A particular strength of our study was that this microarray simultaneously uses a genus-specific probe and species-specific probe to detect targeted bacteria. In recent years, DNA microarrays have been developed to identify bacteria in lung diseases, but they can detect no more than two target genes: one species-specific gene [20] and one conserved gene, including rDNA genes and several phylogenetically conserved genes [11, 12, 21]. For the former, the number of detected bacteria is limited in a single test. For the latter, a single marker cannot achieve the unambiguous detection of closely related or distant species [22]. Therefore, the use of conserved bacterial genes combined with species-specific genes is necessary for the accurate diagnosis of bacteria. To the best of our knowledge, there are no other assays simultaneously using 16S rDNA and bacterial species-specific genes for bacterial identification. Moreover, even when samples that contained bacteria not belonging to the fifteen target bacteria were analysed in this study, they could be identified at the genus level. This method might be a useful addition to the microarray technique.

Furthermore, this microarray could allow rapid bacterial identification directly from patient samples. First, the entire experimental procedure for this assay, from sample receipt to results dissemination, can be completed within 6 h. This is much faster than current methods, most of which require an additional 18–24 h for the growth of bacteria in clinical practice. Second, these fifteen target bacteria cover the most common bacterial causes of community acquired pneumonia (CAP) and hospital acquired pneumonia (HAP) [23, 24], especially atypical pathogens, which are difficult to identify because of lengthy and complicated culture methods [25, 26]. Finally, due to the high-throughput characteristics of the microarray, our microarray can simultaneously detect 15 pathogenic bacteria in one test. These timely and abundant identification results can facilitate the early administration of antimicrobial therapy for pneumonia and prevent bacterial resistance caused by empirical antibiotic therapy. This microarray is worthy of being recommended for use in clinical applications.

This assay was validated with 19 type strains, 119 clinical isolates belonging to 15 target species, 4 clinical isolates belonging to 3 nontarget species and 8 mixed mock specimens. Bacterial strains were cultured overnight in 5 ml of species-specific culture medium and at the corresponding growth temperature. All cells were collected for DNA extraction, and 2.5 μL of the DNA template was used for PCR in microarray validation. This number must be translated into the corresponding number of bacteria since a correction factor has to be introduced due to the extraction efficiency and sample dilution [21]. However, based on the correct identification of the 19 type strains, 119 clinical isolates belonging to 15 target species, 8 mixed mock specimens and 4 clinical isolates belonging to 3 nontarget species, the sensitivity and specificity were both 100%, and the microarray could be concluded to be an efficient diagnostic method for clinical isolates. The criteria for the selection of clinical isolates belonging to nontarget species in this study were that they are often detected in respiratory tract specimens but in most cases are not the main pathogenic bacteria. We used only 4 clinical isolates belonging to 3 nontarget species, which is a small number. Nevertheless, the detection was found to be specific for the 19 type strains, 119 clinical isolates belonging to 15 target species, and 151 bacterial pathogens belonging to 10 target species in clinical samples, and this assay did not detect any of the 4 clinical isolates belonging to nontarget species and the 40 bacterial pathogens belonging to 8 nontarget species in clinical samples. We cannot exclude the possibility that other bacterial species in respiratory tract specimens would cause a reaction with the selected probes, thus interfering with detection. However, this probability is low given the very few cross-reactions observed for the 19 type strains, 123 clinical isolates and 191 bacterial pathogens in the clinical samples tested here.

In this study, the microarray results were compared with the culture results when the microarray effectiveness was assessed with clinical specimens. First, culture is still the most popular method and the gold standard for the identification of bacteria in clinical practice, even though it can produce both false-negative and false-positive results. Second, the 157 clinical specimens tested in our study were collected before antibiotic therapy. Antibiotic therapy can reduce the bacterial burden and viability, potentially leading to negative culture results [27]. Moreover, 121 out of the 157 specimens were endotracheal aspirates and BALF specimens, which are often of better quality than expectorated sputum specimens [28, 29]. Therefore, these procedures prevented the occurrence of false negatives and false positives during bacterial culture to a certain degree. Third, a sequencing method was used to confirm the results when the culture and microarray results were discordant. In this study, the culture and microarray results were different for only one sputum sample. The culture result for this sample corresponded to both *Staphylococcus aureus* and *Acinetobacter baumannii*, whereas the microarray result showed only *Acinetobacter baumannii*, and the results of three replicates of PCR targeting the specific *nuc* gene of *Staphylococcus aureus* were negative. Thus, no specimens were sequenced. Finally, among the 15 bacterial species included in the microarray, 10 different species were found in the clinical samples, which are all relatively easy to identify by culture. Hence, this microarray method was compared with the conventional culture method.

The array was further assessed for its effectiveness in 157 clinical specimens from different patients. Polybacterial infections were well detected in 41 samples. Compared with the culture results, the specificity and sensitivity of the microarray were 100 and 99.4%, respectively. An increased sensitivity of molecular methods based on PCR is reported [13, 30–32]. In this study, only *Staphylococcus aureus* in one sample was not detected by the microarray. The lower sensitivity might be attributable to the DNA extraction procedure or erroneous culture identification. In a recent study, in addition to the standard automated extraction protocol, the addition of proteinase K and lysostaphin was necessary for the efficient extraction of *Staphylococcus aureus* DNA from sputum samples, particularly mucopurulent samples [8, 33]. Unfortunately, no stored specimens could be re-extracted or re-cultured because all the specimens were used in the molecular analyses. Another reason for the lower sensitivity might be that the number of *Staphylococcus aureus* cell was sufficient for culture but was too low for detection with the microarray. The last reason was that clinical specimens did not cover all fifteen target bacteria, especially atypical pathogens, which are difficult to culture. These 5 bacterial species may not have been found in these specimens because the 157 clinical specimens came from the intensive care unit for Pulmonary and Critical Care Medicine. The 5 species were *Mycoplasma pneumoniae, Haemophilus influenzae, Enterobacter cloacae, Legionella pneumophila,* and *Chlamydia pneumoniae,* most of which are difficult to culture. Our DNA microarray would present obvious advantages in detecting these bacteria.

One of the weaknesses of this microarray is that it cannot differentiate between colonization and infection, similar to many other molecular amplification tests. Although some reports have indicated that the quantitative detection of pathogenic bacteria could help to distinguish colonization from infection [8, 34], a meta-analysis showed that clinical outcomes were similar regardless of whether cultures were performed quantitatively or semiquantitatively [35]. Therefore, the identification of the causative agents of infections in patients with pneumonia remains a challenge for clinical microbiology laboratories. Nevertheless, taking the shortened turn-around time and the high throughput of this technique into account, this assay can be concluded to be superior to culture methods.

## Conclusions

In conclusion, this DNA microarray for detecting important bacterial causes of pneumonia has the potential to be used as a faster diagnostic tool than current standard methods. Accurate and timely identification directly from clinical specimens should improve patient management and prevent inappropriate antibiotic therapy.

## Methods

### Study design

First, we designed and evaluated the primers and probes for the target genes and fabricated the microarray. Second, the detection limit of this microarray was evaluated by using a series of 10-fold dilutions (10^1^ copies/μL to 10^6^ copies/μL) of recombinant plasmids. Third, the accuracy of this microarray was evaluated by using genomic DNA from 19 standard strains and 123 clinical isolates (Table 2). Subsequently, 8 mixtures with two or three of these genomic DNAs were randomly mixed and used as templates to assess the ability of this microarray to distinguish mixed pathogens. Finally, the sensitivity and specificity of the microarray were evaluated with clinical samples. Spontaneous sputum specimens, endotracheal sputum aspirate specimens and bronchoalveolar lavage fluid (BALF) specimens were collected in our Pulmonary and Critical Care Medicine department. At the same time, the culture and identification of pathogens were performed in a blinded manner in the Department of Microbiology in our hospital. Direct DNA sequencing was used to confirm the results when they were discordant.

### Specimen collection and processing

The 19 standard strain DNA samples and the 123 clinical isolates used in this study were obtained from the Beijing Institute of Radiation Medicine and Chinese PLA General Hospital (Table 2). All 142 bacterial strains were cultured overnight in 5 ml of species-specific culture medium at the corresponding growth temperature. Genomic DNA of the cells was extracted by boiling with the same volume of lysate buffer (25 mmol/L NaOH, 0.1 nmol/L EDTA, 10 mmol/L Tris-HCl, 1% NP40, 2% Chelex-100, 1% Triton X-100) for 10 min, followed by centrifugation for 2 min at 12000 rpm, absorption of the supernatant and storage at − 70 °C for testing [36]. 16S rDNA was used as a control in the multiplex PCR assay to ensure the standardization and adequacy of the DNA templates from bacteria.

The 157 participating patients with clinically and radiologically confirmed pneumonia came from the intensive care unit of Pulmonary and Critical Care Medicine. All 36 spontaneous sputum specimens, 98 endotracheal sputum aspirate specimens, and 23 bronchoalveolar lavage fluid (BALF) specimens were collected between July 2013 and October 2014. All the specimens were immediately stored at − 70 °C for DNA extraction. At the same time, the culture and identification of the pathogens were performed in a blinded manner at the Department of Microbiology in our hospital. Sputum samples were inoculated onto blood agar plates, chocolate agar plates and MacConkey agar plates using standard techniques and incubated at 37 °C under 5% carbon dioxide in air for 18–24 h. Then, the isolates were identified according to colonial morphology, standard biochemical methods, VITEK-2 analysis (bioMérieux), or matrix-assisted laser desorption ionization-time of flight mass spectrometry. All serum samples were collected and immediately refrigerated at 4 °C for immunoglobulin M antibody assays of *Mycoplasma pneumoniae, Legionella pneumophila,* and *Chlamydia pneumoniae*. An immunoglobulin M antibody detection kit (VIRCELL, Granada, Granada, Spain) was used according to the manufacturer’s instructions, and the results were read under a EUROStar II immunofluorescence microscope (EUROIMMUN, Hanseatic City of Lubeck, Schleswig-Holstein, Germany).

The genomic DNA of the 157 clinical specimens was extracted via the following protocol: 30 min of liquefaction with 4% NaOH, 10 min of boiling of 50 μl of the liquefied specimens with 50 μl of lysate buffer (25 mmol/L NaOH, 0.1 nmol/L EDTA, 10 mmol/L Tris-HCl, 1% NP40, 2% Chelex-100, 1% Triton X-100), 2 min of waiting after addition to the DNA adsorption column, 1 min of centrifugation at 12,000 rpm, washing 2 times with 600 μl of 75% alcohol, and elution in 50 μl of ddH_2_O [36]. All genomic DNAs were stored at − 70 °C until use. We used 10 ng of each DNA template in the multiplex PCR assays to ensure the adequacy of the DNA templates. Additionally, 16S rDNA was included in the multiplex PCR assays as a control to ensure the standardization and adequacy of the DNA templates.

### Construction of reference plasmids

The standard strain DNAs listed in Table 1 were used to construct the reference plasmids. Plasmids containing the target genes were generated by cloning the PCR products with the pMD18™-T vector system (TaKaRa, Shiga, Japan). All plasmids were defined by sequencing. Plasmid extracts were diluted in ddH_2_O to 10^6^ copies/μL in a tenfold dilution series for use in microarray optimization.

### Primer and probe design and evaluation

We selected both 16S rDNA and 15 bacterial-specific genes as target genes to identify bacteria at the species level. The 15 bacterial-specific genes were *lytA* of *Streptococcus pneumoniae, nuc* of *Staphylococcus aureus, P6* of *Haemophilus influenzae, phoA* of *Escherichia coli, mdh* of *Klebsiella pneumoniae, toxA* of *Pseudomonas aeruginosa, gltA* of *Acinetobacter baumannii, P1* of *Mycoplasma pneumoniae, ddl* of *Enterococcus faecalis* and *Enterococcus faecium, dnaJ* of *Enterobacter cloacae, chitA* of *Stenotrophomonas maltophilia, recA* of *Burkholderia cepacia, mip* of *Legionella pneumophila* and *ompA* of *Chlamydia pneumoniae.* All gene sequences were downloaded from NCBI (http://www.ncbi.nlm.nih.gov/genomes). A pair of universal primers was designed to amplify specific sequences in conserved upstream and downstream regions of the 16S rDNA sequence. In the variable regions between universal primers, specific probes and a positive control probe were designed. *Klebsiella pneumoniae*, *Enterobacter cloacae* and *Escherichia coli* were detected with the same specific probe because of their highly similar sequences. For the 15 bacterial-specific genes, we designed the primers and probes using DNAMAN 6 and Oligo 7 software, respectively. Primers were selected in conserved upstream or downstream regions, and probes were designed in the variable portion of the sequences. All primer and probe sequences were aligned using BLAST (http://blast.ncbi.nlm.nih.gov/) to compare the homology between potential targets belonging to the same genus. To evaluate the efficiency of all primers, reference genomic DNAs of the 15 bacterial species were amplified and examined by 2% agarose gel electrophoresis. All primers and probes were finally confirmed by the sequence analysis of the PCR products from the reference plasmids.

### Microarray preparation

This DNA microarray was designed to contain 32 probes, including 1 universal 16S rDNA probe and 3 negative control probes, in eight columns and eight rows. The universal 16S rDNA probe was used to detect whether the samples contained bacteria. The probes were synthesized by Sangon Biotech Co., Ltd. (Shanghai). Each probe (50 μM final concentration) was spotted twice repeatedly with a noncontact Nanoplotter 2.1 inkjet (GeSim, Dresden, Germany) onto the aldehyde chip after mixing with uniform proportional printing buffer (5% glycerol, 0.1% sodium dodecyl sulphate (SDS), 6× saline sodium citrate buffer (SSC), and 2% (wt/vol) Ficoll 400). The microarray layout is shown in Fig. 1a. Microarrays were prepared as previously described by our research group [37].

### Multiplex asymmetric PCR

The primers for 16S rDNA and the 15 specific genes were divided into three groups for multiplex asymmetric PCR. Reactions were carried out on a Veritil 96-well Thermal Cycler instrument (Applied Biosystems by Life Technologies, Singapore). The final reaction volume for each multiplex asymmetric PCR assay was 25 μl, including the same Multiplex PCR 5× Master Mix reagents (5 μl, New England Biolabs, UK) and amount of DNA template (2.5 μl). The forward and reverse primer concentrations for 16S rDNA, *P6* and *mip* were 0.08  μM and 0.4  μM, respectively. For the other targets, these concentrations were 0.16  μM and 0.8  μM, respectively. The cycling parameters were optimized as follows: 10 min at 95 °C; 35 cycles of 30 s at 95 °C, 30 s at 55 °C, and 1 min at 68 °C; and a final extension of 5 min at 68 °C.

### Hybridization and signal detection

Prior to hybridization, the PCR products were denatured at 98 °C for 5 min and chilled on ice. A 2.5 μl aliquot of each amplification product from the three multiplex PCR assays was mixed with 7.5 μl of hybridization buffer (0.6% SDS, 10% formylamine, 8× SSC, and 10× Denhardt). A total of 15 μl of the hybridization mixture was reacted with the probes at 45 °C for 1 h. Thereafter, the slide was washed for 1 min each with washing buffer A (1× SSC and 0.2% SDS), washing buffer B (0.2× SSC), and washing buffer C (0.1× SSC) for and then dried by centrifugation. Subsequently, 1:1500-diluted streptavidin-horseradish peroxidase (HRP) was incubated in each reaction chamber on the chip for 30 min at 37 °C, and the slide was washed once with PBST (0.05% Tween 20) for 1 min and dried by centrifugation. Finally, the regions of hybridization on the slide were covered with 20 μl phospho-tyrosine (Millipore, USA), and the signal was immediately detected with a portable biochip chemiluminescence imaging instrument (Academy of Military Medical Sciences, China).

## Supplementary information

**Additional file 1: Figure S1.** PCR products examined by 2% agarose gel electrophoresis. **a**, Agarose gel electrophoresis of PCR products amplified using the universal 16S rDNA primer. DNA templates were extracted from: 1 *ddH*_*2*_*O*; 2 *Haemophilus influenzae* (ATCC9007); 3 *Haemophilus influenzae* (ATCC33533); 4 *Staphylococcus aureus*; 5 *Acinetobacter baumannii*; 6 *Escherichia coli*; 7 *Streptococcus pneumoniae*; 8 *Pseudomonas aeruginosa*; 9 *Chlamydia pneumoniae*; 10 *Mycoplasma pneumoniae*; 11 *Legionella pneumophila*; 12 *Klebsiella pneumoniae*; 13 *Enterococcus faecalis*; 14 *Enterococcus faecium*; 15 *Stenotrophomonas maltophilia*; 16 *Burkholderia cepacia*; 17 *Enterobacter cloacae*; respectively. **b**, Agarose gel electrophoresis of PCR products amplified using 15 pairs of primers for the 15 bacterial specific genes. The 15 bacterial specific genes were 1 *P1*; 2 *ddl (for Enterococcus faecalis)*; 3 dnaJ; 4 *mdh*; 5 *chitA*; 6 *lytA*; 7 *recA*; 8 *phoA*; 9 *ddH*_*2*_*O*; 10 *ddl (for Enterococcus faecium)*; 11 *gltA*; 12 *mip*; 13 *nuc*; 14 *toxA*; 15 *ompA*; 16 *P6*, respectively.

## Data Availability

The data used and analysed for the current study are available upon request from the first author Xiuqing Ma (E-mail: mxq820812@163.com).

## Abbreviations

PCR: Polymerase chain reaction
rDNA: Ribosomal DNA
ATCC: American Type Culture Collection
CMCC: National Center for Medical Culture Collections
CGMCC: China General Microbiological Culture Collection Center
CAP: Community acquired pneumonia
HAP: Hospital acquired pneumonia
BALF: Bronchoalveolar lavage fluid
SDS: Sodium dodecyl sulfate
SCC: Salinesodium citrate buffer
HRP: Streptavidin-horseradish peroxidase
