# Supplementary figures and images for: Development of a DNA microarray assay for rapid detection of fifteen bacterial pathogens in pneumonia
Source: BMC Microbiol. 2020 Jun 23;20:177. doi: 10.1186/s12866-020-01842-3 (PMC7310556; doi:10.1186/s12866-020-01842-3)

**a**

1 2 3 4 5 6 7 8 9 10 11 12 13 14 15 16 17


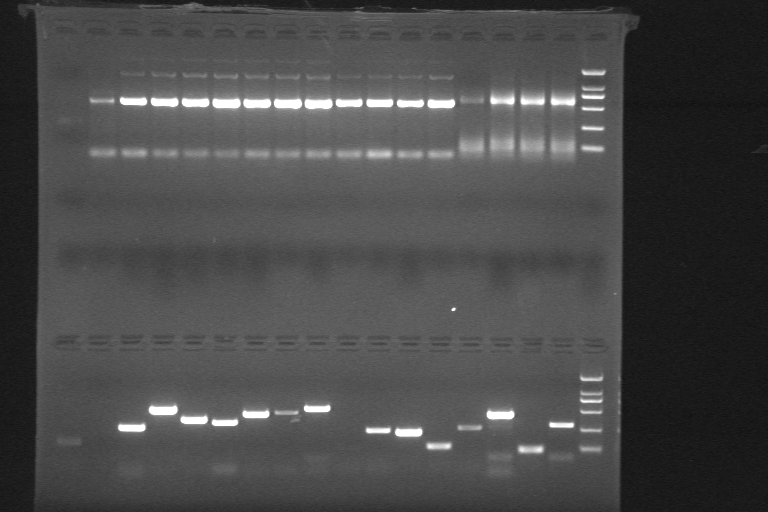


750

1000

500

2000

250

100

**b**


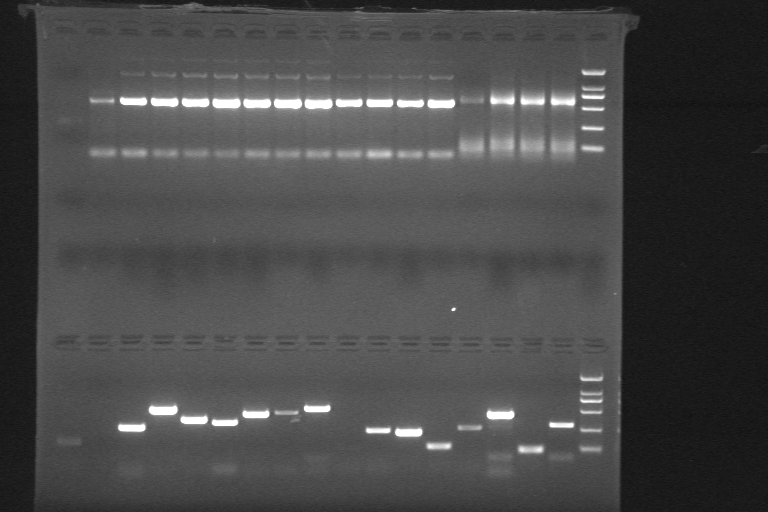


2000

1000

750

500

100

250

1 2 3 4 5 6 7 8 9 10 11 12 13 14 15 16

Supplement: Supplementary file 1 — Additional file 1: Figure S1. PCR products examined by 2% agarose gel electrophoresis. a, Agarose gel electrophoresis of PCR products amplified using the universal 16S rDNA primer. DNA templates were extracted from: 1 ddH2O; 2 Haemophilus influenzae (ATCC9007); 3 Haemophilus influenzae (ATCC33533); 4 Staphylococcus aureus; 5 Acinetobacter baumannii; 6 Escherichia coli; 7 Streptococcus pneumoniae; 8 Pseudomonas aeruginosa; 9 Chlamydia pneumoniae; 10 Mycoplasma pneumoniae; 11 Legionella pneumophila; 12 Klebsiella pneumoniae; 13 Enterococcus faecalis; 14 Enterococcus faecium; 15 Stenotrophomonas maltophilia; 16 Burkholderia cepacia; 17 Enterobacter cloacae; respectively. b, Agarose gel electrophoresis of PCR products amplified using 15 pairs of primers for the 15 bacterial specific genes. The 15 bacterial specific genes were 1 P1; 2 ddl (for Enterococcus faecalis); 3 dnaJ; 4 mdh; 5 chitA; 6 lytA; 7 recA; 8 phoA; 9 ddH2O; 10 ddl (for Enterococcus faecium); 11 gltA; 12 mip; 13 nuc; 14 toxA; 15 ompA; 16 P6, respectively. [file 12866_2020_1842_MOESM1_ESM.docx]
